# High density mapping guided partial antral ablation for a pulmonary vein isolation

**DOI:** 10.1038/s41598-021-96004-4

**Published:** 2021-08-16

**Authors:** Jongmin Hwang, Seongwook Han, Chun Hwang, Hyoung-Seob Park, Cheol Hyun Lee, In-Cheol Kim, Yun-Kyeong Cho, Jin Wook Chung, Hyuck-Jun Yoon, Hyungseop Kim, Chang-Wook Nam, Seung-Ho Hur, Jin Young Kim, Yun Seok Kim, Woo Sung Jang

**Affiliations:** 1 Division of Cardiology, Department of Internal Medicine, Keimyung University Dongsan Hospital, Keimyung University School of Medicine, 1035 Dalgubeol-daero Dalseo-gu, Daegu, 42601 Republic of Korea; 2 Cardiology, Revere Health, Provo, UT USA; 3 Department of Radiology, Keimyung University Dongsan Hospital, Keimyung University School of Medicine, Daegu, Republic of Korea; 4 Department of Thoracic and Cardiovascular Surgery, Keimyung University Dongsan Hospital, Keimyung University School of Medicine, Daegu, Republic of Korea

**Keywords:** Interventional cardiology, Atrial fibrillation

## Abstract

The muscular discontinuities at the pulmonary vein (PV)–left atrial (LA) junction are known. The high-density mapping may help to find the muscular discontinuity. This study evaluated the efficacy of a partial antral ablation for a pulmonary vein (PV) isolation using high density (HD) mapping. A total of 60 drug-refractory atrial fibrillation (AF) patients undergoing catheter ablation were enrolled. The detailed activation mapping of each PV and LA junction was performed using an HD mapping system, and each PV segment’s activation pattern was classified into a “directly-activated from the LA” or “passively-activated from an adjacent PV segment” pattern. The antral ablations were performed at the directly-activated PV segments only when the PV had “passively-activated segments”. If the PV did not contain passively-activated segments, a circumferential antral ablation was performed on those PVs. A “successful partial antral ablation” was designated if the electrical isolation of targeted PV was achieved by ablation at the directly-activated segments only. If the isolation was not achieved even though all directly-activated segments were ablated, a “failed partial antral ablation” was designated, and then a circumferential ablation was performed. Among 240 PVs, passively-activated segments were observed in 140 (58.3%) PVs. Both inferior PVs had more passively-activated segments than superior PVs, and the posteroinferior segments had the highest proportion of passive activation. The overall rate of successful partial antral ablation was 85%. The atrial tachyarrhythmia recurrence was observed in 10 patients (16.7%) at 1-year. HD mapping allowed the evaluation of the detailed activation patterns of the PVs, and passively-activated segments may represent muscular discontinuity. Partial antral ablation of directly-activated antral segments only was feasible and effective for a PVI.

## Introduction

The electrical isolation of the pulmonary veins (PVI) is the cornerstone of the current ablation strategies for atrial fibrillation (AF) treatment because the PVs are the most common trigger site for AF^1^. A wide bi-antral circumferential ablation (WACA) is more effective than a segmental PV isolation in achieving freedom from atrial tachyarrhythmia recurrence during the long-term follow-up. Therefore, it is widely accepted as an initial ablation strategy^2^. However, the WACA technique requires a much larger number of ablation applications and higher energy to achieve a complete isolation^3^. Furthermore, atrial muscle injury by extensive ablation can deteriorate the atrial function such as the contraction and compliance^4^. On the other hand, muscular discontinuities and abrupt changes in the fiber orientation in the human PV–left atrial (LA) junction have been previously reported, and an electrical PV isolation can usually be achieved without a complete circumferential ablation^5,6^. However, the prior electroanatomical mapping (EAM) systems have a limitation in understanding the complex relationship of the PV–LA junction due to the relatively low resolution.

A recently developed high-density (HD) EAM coupled with an automatic annotation algorithm and smaller, closely spaced multielectrode catheters has allowed the rapid and accurate identification of critical isthmuses and low-voltage regions of interest. A detailed mapping around the PV–LA junction by the HD EAM system may help find the site of muscular discontinuity and thus avoid any unnecessary ablation for the PVI.

In this study, we hypothesized that an accurate identification of the activation pattern at the PV–LA junction using the HD mapping system and a partial antral ablation based on this finding could allow for a complete electrical isolation of the PVs without a circumferential antral ablation.

## Methods

### Study population

The patients who received a first radiofrequency catheter ablation (RFCA) for drug-refractory paroxysmal or persistent AF at our hospitals were included. This study was a single center, prospective study and consecutively enrolled 60 patients between October 2018 and March 2019. In these patients, a partial antral ablation was performed for the PVI under HD mapping with the Rhythmia three-dimensional (3D) EAM system (Rhythmia HD system, Boston Scientific, Cambridge, MA). All patients signed their informed consent for the procedure, and the study was approved by the Institutional Review Board of Keimyung University Dongsan Hospital. All experiments were performed in accordance with relevant guidelines and regulations.

### General ablation procedure

The RFCA and mapping procedures were performed under a fasting state with conscious sedation after withdrawal from anti-arrhythmic drugs for a period equal to five-times the half-lives of the drugs. Three multipolar catheters were inserted through the left femoral region and placed in the right atrium, coronary sinus, and His bundle. Two 8.5 Fr non-steerable long sheaths (Swartz Braided Transseptal Guiding Introducer SL1, Abbott, St. Paul, MN) were placed in the right atrium through the right femoral region and inserted into the LA using transseptal punctures. After the transseptal puncture, an activated clotting time of over 350 s was maintained with intermittent intravenous heparin boluses.

The definition of the PV antrum was defined as the area ≥ 1.5 cm away from the PV ostium as identified by PV angiography and a 3D EAM reconstruction^2^. However, because there are high inter-individual variations in the anatomy of the PV antrum, the ablation line along the PV antrum was predesigned and adjusted differently for each patient. In general, the ablation line was designated to be 5–10 mm toward the LA from the point of the fusion of the PV and LA potentials. The PV antral electrograms were confirmed by the ablation catheter tip during the sequential positioning of a catheter at multiple sites along the ablation line. The method of ablation was a continuous dragging of the catheter tip along the predesigned ablation line, and the energy delivery was continued until the maximum local amplitude decreased by ≥ 75% or to < 0.1 mV. To protect the esophagus, the esophagus was reconstructed and merged with the heart and surrounding structures before the procedure to confirm its approximate location. With reference to that, the real-time location of the esophagus was monitored through intracardiac echocardiography during the procedure. When ablation was required over the esophagus, the minimum ablation energy required for an electrogram reduction was applied.

The endpoint of the PVI was entrance block of the PV identified by a multipolar catheter. Over 20 min after the acute electrical isolation of the PVs, acute PV reconnections and dormant PV connection were tested under an isoproterenol infusion. If the patient had a history of typical atrial flutter or typical flutter was induced during the procedure, a cavo-tricuspid isthmus ablation was additionally performed. However, additional substrate modification was not performed.

### Methods of partial antral ablation

**Figure 1:**
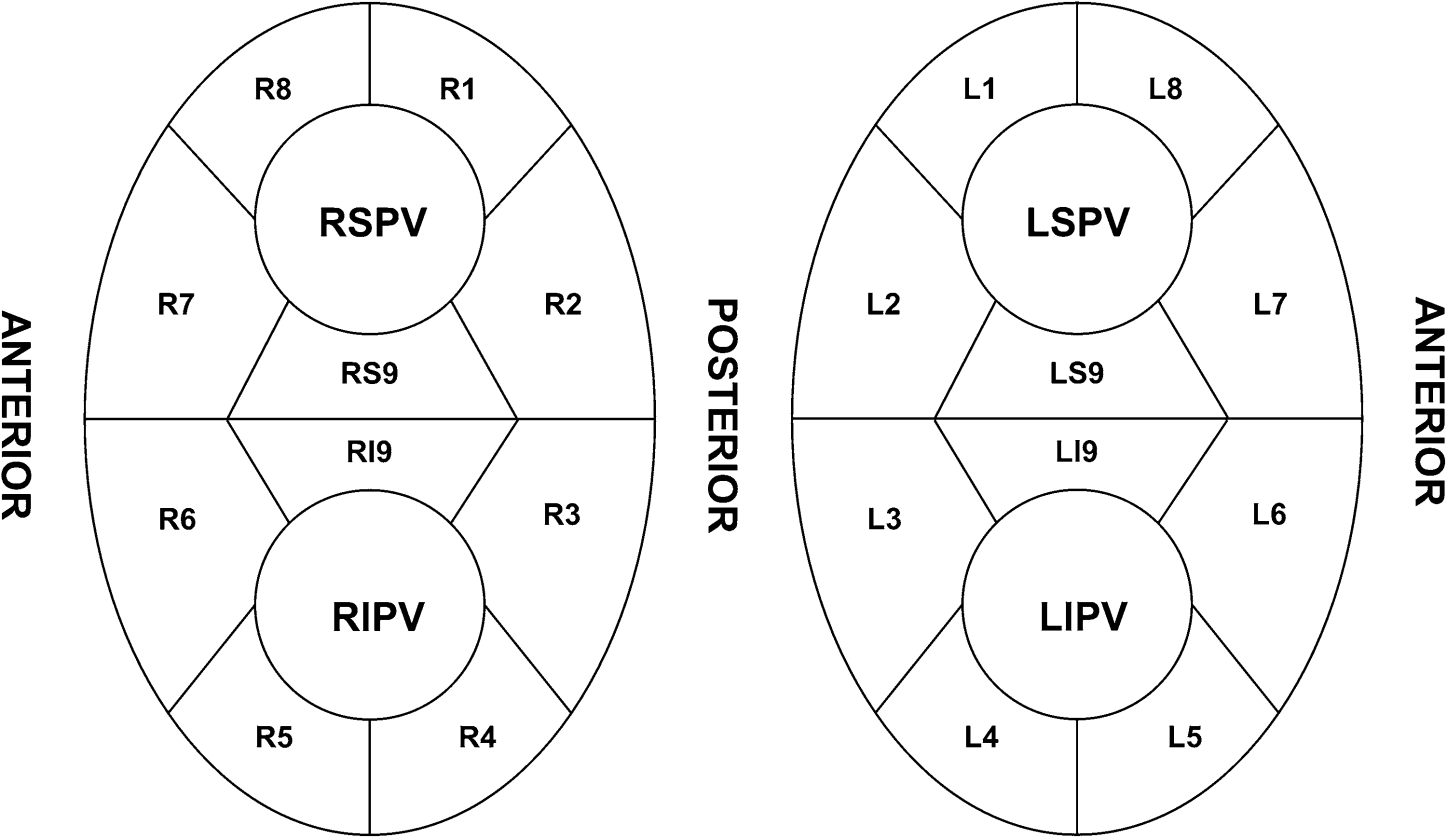
Segments of the PVs. The left and right pulmonary veins are divided into 10 segments, respectively. The carinas are divided into superior and inferior segments.

The LA geometry and activation mapping were obtained with a 64-pole basket mapping catheter (IntellaMap Orion, Boston Scientific, Cambridge, MA) during mid-coronary sinus (CS) pacing with a cycle length of 600 ms. The sinus rhythm was achieved by the internal DC cardioversion if the presenting rhythm was AF. We enrolled the patients whose rhythm was maintained in the sinus during mapping. The beat acceptance criterion was regulated by the following six components, which were related to the stability: cycle length (< 10 ms), propagation reference (< 5 ms), respirations (< 10–15 μV), mapping catheter movement (< 1 mm), electrogram stability (< 25%), and tracking quality (< 3 mm); the mapping points were automatically acquired when all these criteria were met. The projection distance and confidence mask were 2 mm and 0.04 mV, respectively. After mapping was completed, the activation patterns around the left- and right-sided PVs and antrum were evaluated by a propagation video. As shown in Fig. 1, the PV antrum was divided into ten segments for each left and right PV, and the activation patterns of each segment were classified into the following two types.

**Figure 2:**
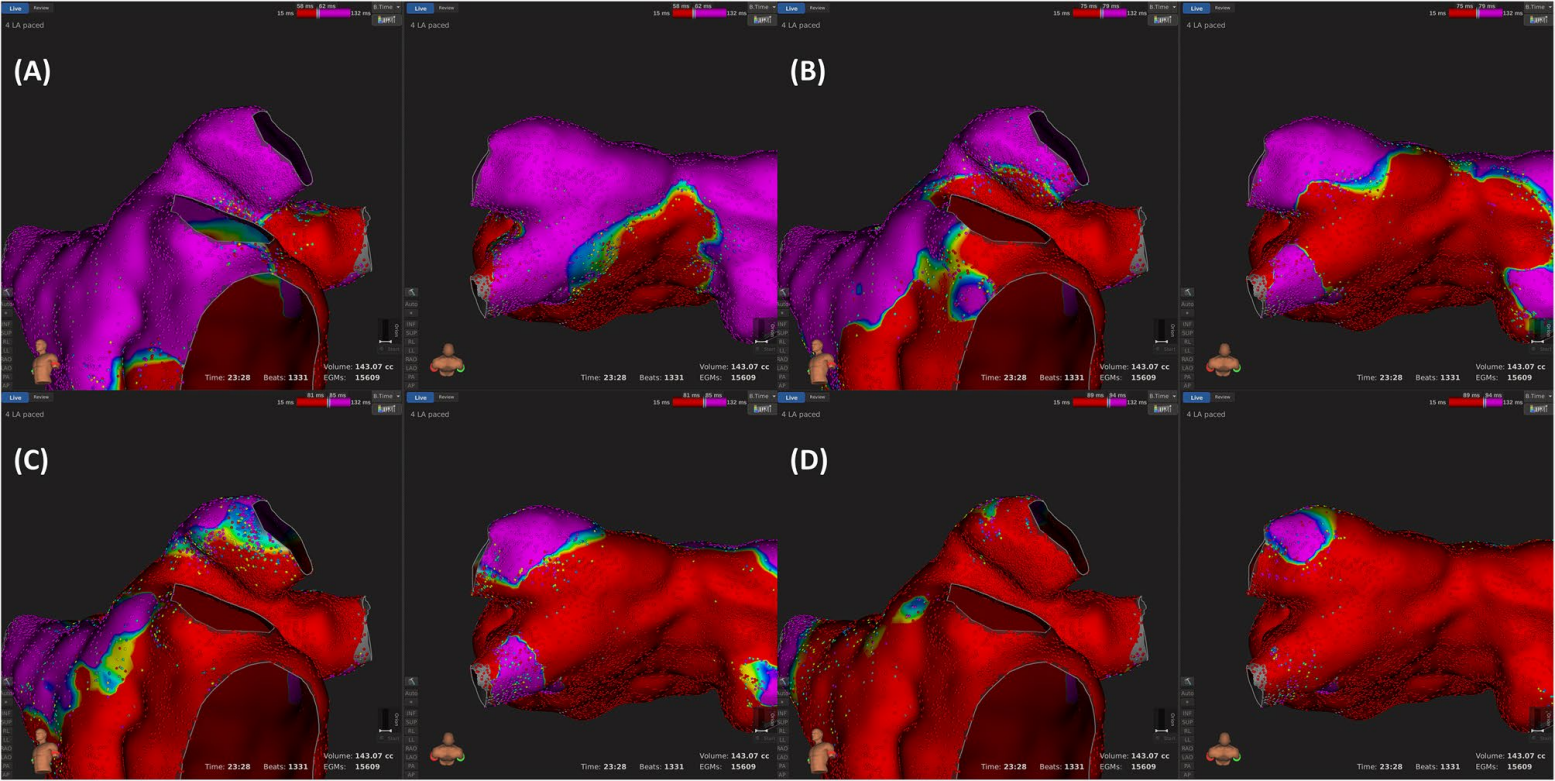
Examples of a left superior pulmonary vein (LSPV) antral activation pattern (from (**A**) to (**D**), sequential according to the time), which shows a “directly activated from the LA” pattern. Activation mapping was performed during coronary sinus pacing. Each sub-figure is comprised of anterior and postero-superior views for the LSPV. (**A**) Most of the LSPV is not activated at this time and the activation is initiated from the ridge and carina. (**B**) After 17 ms, the antero-inferior segment, carina, and postero-inferior segment are activated first. (**C**) After 6 ms, the remaining postero-superior and antero-superior segments are activated. (**D**) After 8 ms, the entire antral level LSPV was activated. The site with the latest activation is the postero-superior PV, which is inside the PV and not at the antral level of the PV.

**Figure 3:**
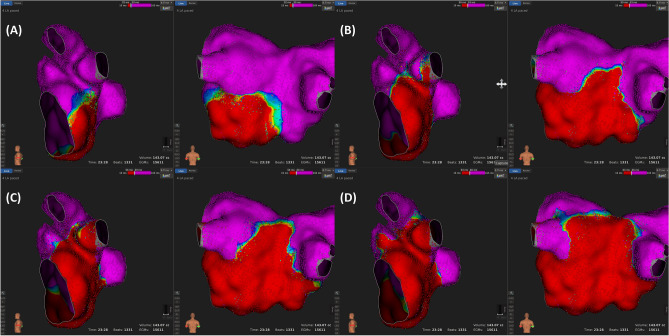
Examples of a left inferior pulmonary vein (LIPV) antral activation sequence according to the time [from (**A**) to (**D**)], which shows the presence of being “passively activated from an adjacent segment”. Activation mapping was performed during coronary sinus pacing. Each sub-figure is comprised of lateral and posterior views of the LIPV. (**A**) Most of the LIPV is not activated at this time, and the activation is initiated from the inferior ridge and inferior segment. (**B**) After 18 ms, the ridge segments are activated first. (**C**) After 6 ms, the carina segment is activated. (**D**) After 7 ms, the posterosuperior segment starts to activate. However, the postero-inferior segment is still not activated.

1. Directly activated from the LA: this type of activation pattern was defined when the segment was activated from the LA without a time delay, and the activation was simultaneous with the adjacent PV segments. Examples of direct activation patterns of the PVs are shown in Fig. 2 and Supplementary video 1.
2. Passively activated from an adjacent PV segment: this type of activation pattern was defined when the segment was not activated from the LA, but from an adjacent PV segment. Activation of these segments started at least 40–45 ms later than the first activated PV segment. Examples of a PV antrum with passively activated segments are shown in Fig. 3, Supplementary video 2A, 2B, 2C, and Supplementary Fig. 1.

**Figure 4:**
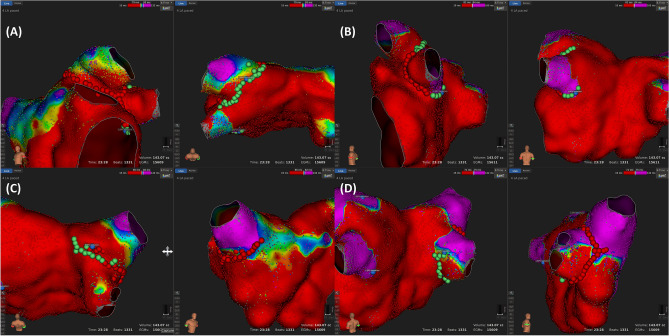
Examples of the ablation points in Figs. 2 and 3 (same patient). In this patient, the left superior pulmonary vein (PV) did not exhibit a passively activated segment. Therefore, a circumferential ablation at the PV antrum was performed (**A**). However, the left inferior (**B**) and right superior (**C**)/inferior (**D**) PVs exhibited passively activated segments, and an electrical isolation of the PVs was obtained only with a partial antral ablation (successful partial antral ablation). The red dots indicate a 30 W ablation. The green dots indicate a 25 W ablation. The blue dots indicate the isolation point.

After each segment’s activation pattern was determined, ablation was initially performed at the PV antrum of the directly-activated segments. The directly-activated segments of the antero-superior/inferior PVs were first ablated (L7-LS9-L8 for the left superior PV [LSPV], L6-LI9-L5 for the left inferior PV [LIPV], R7-RS9-R8 for the right superior PV [RSPV], and R6-RI9-R5 for the right inferior PV [RIPV]) where the muscle layer is known to be thicker than the other segments based on the previous study results^7^. If PVI was not achieved by directly-activated segments of the antero-superior/inferior PV, then the directly-activated segments of the postero-superior/inferior PV were ablated. A “successful partial antral ablation” was designated if the PVI was achieved by ablation at the directly-activated segments only. If the PVI was not achieved even though all directly-activated segments were ablated, a “failed partial antral ablation” was designated, and then a circumferential ablation was performed in that PVs. If the PV did not contain passively-activated segments, a circumferential antral ablation was performed on those PVs. Examples of ablation according to the activation pattern are shown in Fig. 4.

The radiofrequency energy was delivered using an irrigated ablation catheter (THERMOCOOL SF Bi-Directional catheter, Biosense Webster, Diamond Bar, CA) in a power-controlled mode using a power of 30 W, upper-limit temperature of 45 °C, and irrigation rate of 17 mL/min for a duration of 25–30 s. Ablation of the posterior wall was performed with a power of 25 W for 15–20 s. The ablation lesions were tagged manually at the operator's discretion.

### Follow-up

All patients were regularly seen in the outpatient clinic after the procedure: 1-week, 1-month, 3-month after the procedure, and every 3-month thereafter. At every visit, the patients received a 12-lead ECG. Holter monitoring was performed at 3-months, 6 months, and 12-months. A detailed history of the patients’ symptoms suggestive of potential arrhythmia recurrences was taken. In the case of undocumented symptoms suspicious of arrhythmia recurrences, documentation by an additional 12-lead ECG or Holter monitoring was performed. A documented symptomatic or asymptomatic atrial tachyarrhythmia episode lasting > 30 s was defined as a recurrence.

### Primary and secondary endpoints

The primary endpoint was the freedom from any atrial tachyarrhythmia recurrences during the 1-year follow-up period. The secondary endpoints were the procedure time, fluoroscopy time, duration of the ablation energy delivery, and procedural complications.

### Statistical analyses

Continuous variables are expressed as the mean value ± standard deviation. Categorical variables are expressed as numbers and percentages. Atrial arrhythmia free survival curves were constructed using Kaplan–Meier estimates and compared with the log-rank test. A 2-tailed *P* value of < 0.05 was considered statistically significant. Baseline statistical analyses were performed using the MedCalc Statistical Software version 19.5.3 (MedCalc Software Ltd, Ostend, Belgium). A survival analysis was performed using Prism 8.0.

## Results

### Baseline demographic and general procedural characteristics

A total of 60 drug-refractory AF patients were included in this study. The baseline and procedural characteristics are summarized in Table 1. The mean age was 57.8 ± 5.8 years and male sex was 50 (66.2%). Patients with paroxysmal AF were 49 (81.7%). The mean LA mapping points were 16,446 ± 3,852. The mean ablation time was 2764.2 ± 860.8 s, and the mean procedure time was 202.9 ± 38.2 min. One patient suffered from a groin hematoma after the procedure. Otherwise, there were no acute or late complications during the follow-up period.

**Table 1:** Demographic and general procedural characteristics.

Characteristic	N = 60
Male	50 (66.2)
Age (y)	57.8 ± 5.8
**Type of AF**	
Paroxysmal	49 (81.7)
Persistent	11 (18.3)
LV EF (%)	61.5 ± 6.3
LA volume (cc)	90.2 ± 25.7
History of hypertension	23 (38.3)
History of diabetes	8 (13.3)
Procedure time (min)	202.9 ± 38.2
Fluroscopy time (s)	1570.1 ± 473.8
Ablation time (s)	2764.2 ± 860.8
AF Recurrence at 1-year	10 (16.7)

Values are presented as the n (%) or mean ± SD. Abbreviations: *AF* atrial fibrillation, *LV* left ventricle, *EF* ejection fraction, *TIA* transient ischemic attack, *LA* left atrium, *SEC* spontaneous echo contrast, *TEE* transesophageal echocardiography.

### Procedural characteristics of the partial antral ablation

**Figure 5:**
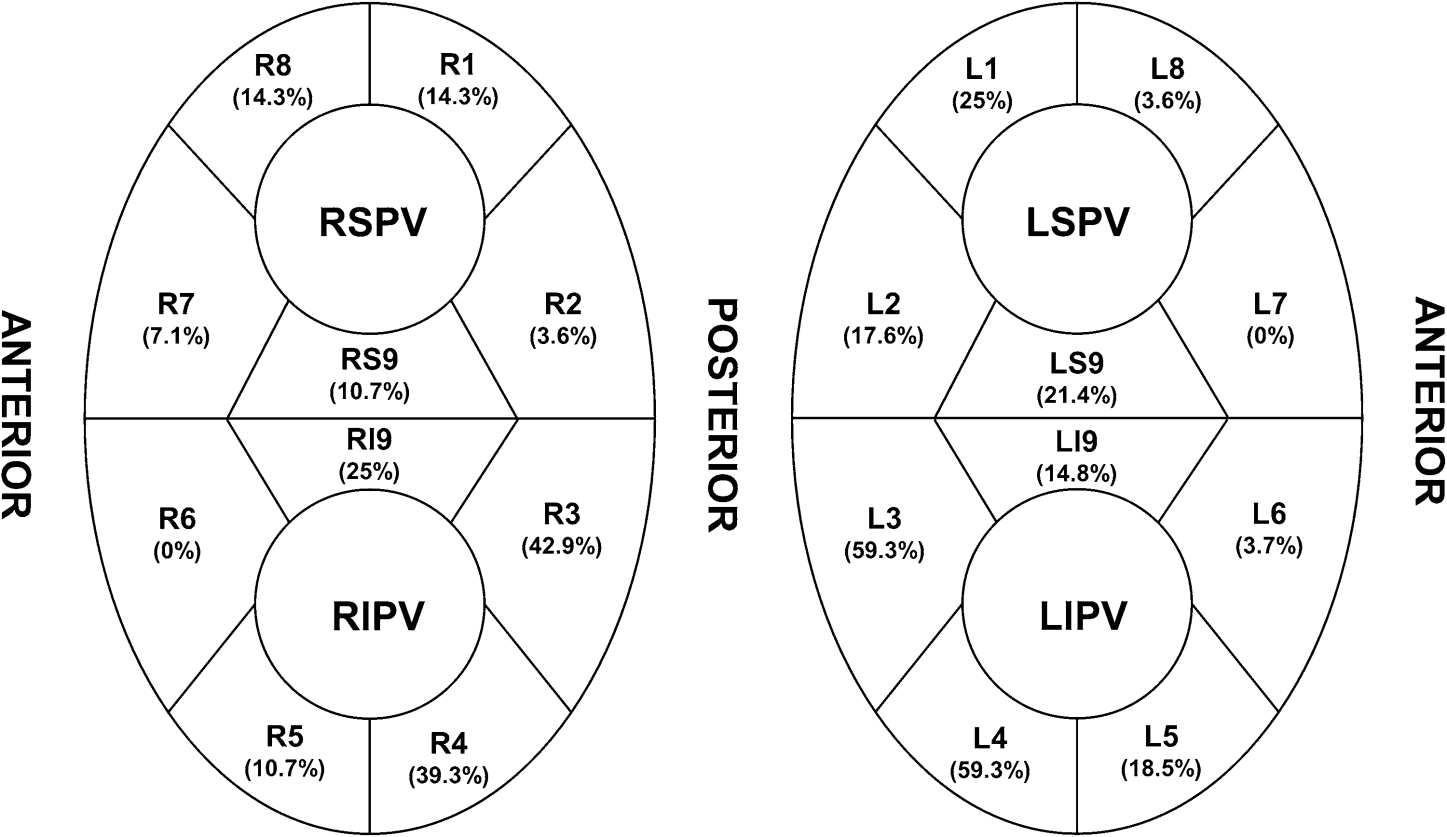
Proportion of passively activated segments existing at each pulmonary vein antrum.

Among 240 PVs, passively-activated segments were observed in 140 (58.3%) PVs. The presence and proportion of passively-activated segments at each PV antrum is summarized in Table 2 and Fig. 5.

**Table 2:** Presence of a passively activated segment and the success rate of a partial antral ablation.

	Passively activated segment	Success rate of partial ablation	*P*-value
LSPV	Present (43.3%, 26/60)	73.1% (19/26)	0.020
	Absent (56.7%, 34/60)	23.5% (8/34)	
LIPV	Present (78.3%, 47/60)	93.6% (44/47)	0.059
	Absent (21.7%, 13/60)	30.7% (4/13)	
RSPV	Present (40%, 24/60)	62.5% (15/24)	0.094
	Absent (60%, 36/60)	27.8% (10/36)	
RIPV	Present (71.7%, 43/60)	95.3% (41/43)	0.014
	Absent (28.3%, 17/60)	23.5% (4/17)	

Values are presented as the n (%) or mean ± SD. Abbreviations: *LSP* left superior pulmonary vein, *LIPV* left inferior pulmonary vein, *RSPV* right superior pulmonary vein, *RIPV* right inferior pulmonary vein.

Both inferior PVs had more passively-activated segments than the superior PVs (superior 50 PVs vs. inferior 90 PVs, *P* < 0.0001), and the posteroinferior segment (L3-4, R3-4 segment) exhibited the highest proportion of passively-activated segments. The success rates of a partial antral ablation are also described in Table 2. The success rates of a partial antral ablation for the PVI were higher when passively-activated segments were present (PV with passively-activated segments: 85% [119/140] vs. PV without passively-activated segments: 26% [26/100], *P* < 0.0001). Especially, both inferior PVs had higher success rates than the superior PVs when passively-activated segments were present (superior 68% [34/50] vs. inferior 94.4% [85/90], *P* < 0.0001). This trend was maintained even when considering each of the four PVs separately. The proportions of ablated segments in the successful partial antral ablation group for each PV were as follows: 80% for the LSVP, 48% for the LIPV, 82% for the RSPV, and 65% for the RIPV).

### Clinical outcomes

Atrial tachyarrhythmia recurrence was observed in 10 (16.7%) patients during the 1-year follow-up period. Three patients recurred with atrial tachycardia and 7 patients recurred with AF. The survival curve graph is shown in Supplementary Fig. 2.

## Discussion

The current HD mapping technology with the Rhythmia HD mapping system allows for evaluating the detailed activation patterns of the PVs. It has revealed passively-activated PV antral segments, which may have represented the segments of the muscular discontinuity of the PV–LA junction. A partial antral ablation strategy, with an ablation of only directly-activated antral segments guided by HD activation mapping, was feasible and effective for the PVI. Further, the 1-year atrial tachyarrhythmia recurrence was not significantly different from that of the currently known AF ablation outcome^8^. By the partial antral ablation technique, a PVI can be achieved by smaller area ablation than the conventional technique. In addition, PVI can be highly achievable in the inferior PVs without posterior wall ablation. Therefore, this technique may provide less chance of collateral damage or complications by catheter ablation.

Although there is still no consensus about the definition of HD mapping, it refers to when more than 400 mapping points and an average of 2000–6000 mapping points are acquired for a given heart chamber^9,10^. HD mapping allows for an improved substrate characterization, better understanding of the arrhythmia mechanism, and better selection of ablation targets in atrial and ventricular arrhythmias^11,12^. Currently, all three 3D mapping systems offer high-density mapping techniques: the Pentaray catheter by Biosense Webster and Carto 3, Advisor HD Grid catheter by Abbott for the EnSite Precision System, and Intellamap Orion catheter by Boston Scientific for the Rhythmia HDx System. The quality of the high-density maps varies according to the different mapping systems^13^. The Intellamap Orion catheter has the highest number of catheter electrodes, smallest surface area of the electrodes, and highest number of automatic mapping points per map with the combination of all these components^10^.

Anatomical and histological studies of the human LA have revealed the complex anatomy and wide variations in the PV–LA junction and PVs^14^. These variations include the gross anatomy and at the histological level, especially the differences in the length, orientation, and thickness of the myocardial fibers extending into the PVs. In 2001, S. Y. Ho et al. already reported in their postmortem histologic study that the myocardial architecture in normal pulmonary veins is highly variable^7^. They found that the sleeves in the superior veins were thickest inferiorly (at 6 o’clock), and thinnest superiorly (at 12 o’clock), whereas the converse arrangement was the case in the inferior veins. Further, the sleeves were significantly thicker in the left superior veins (*P* < 0.05), but not in the right veins. Cabrera et al. reported an intravascular ultrasound study of postmortem pulmonary veins and found that there are examples showing the lack of an intermediate layer attributable to the absence of myocardial extensions into the pulmonary vein^15^. Tan et al. found that muscular discontinuities and abrupt fiber orientation changes are present in > 50% of PV–LA segments, which are presumed to create significant substrates for re-entry^6^. All of these findings provided the basis for the reason why the HD mapping could reveal the passively-activated segments. Saito et al. reported that the alignment of the myocardial fibers within the myocardial sleeves varies considerably, and non-uniform patterns in which the myocardial fibers show an oblique course with crossovers are more frequent^16^. Interestingly, they also reported that the myocardial sleeves were longer at the superior PVs than inferior PVs, which is thought to be in line with our findings that there were more passively-activated segments in the inferior PVs.

Currently, circumferential PV isolation remains the “gold standard” and the recommended approach for catheter ablation of AF. However, as described above, the discontinuity in the muscular sleeves and non-uniformity in the muscle thickness at the antral level raise questions of whether this is the only ablation strategy that can be applied to all patients. Therefore, to bridge the gap between the knowledge and practice, we performed a detailed activation mapping of each PV. Our study findings indicated that there are passively-activated PV segments, which may represent the muscular discontinuity of the PV–LA junction. In addition, in most PVs with passively-activated segments, a PVI could be obtained even when only directly-activated segments were ablated. The clinical significance of these results is as follows. First, performing a partial antral ablation can reduce the amount of ablation needed to obtain a complete PVI, thus minimizing the myocardial damage. This would be helpful for AF patients whose atrial cardiomyopathy is in the early stage when considering AF is progressive. Second, the partial antral ablation, especially sparing the posterior segments of the inferior veins from ablation, may reduce the chance of collateral damage to the esophagus or descending aorta, which is one of the major complications of AF RFCA.

In our study, a partial antral ablation was not successful for all PVs with passively-activated segments (success rates of a partial antral ablation when passively-activated segments were present: 73% of LSPVs, 63% of RSPVs, 94% of LIPVs, and 95% of RIPVs). Further, a partial antral ablation was successful at some PVs without passively activated segments (success rates of a partial antral ablation when no passively-activated segments were present: 24% of LSPVs, 31% of LIPVs, 28% of RSPVs, and 24% of RIPVs). There are several possible reasons why the PVI did not correlate with the ablation strategies based on the presence of passively-activated segments. It has already been reported that the activation pattern and low voltage areas in the LA and LA-PV junction may change depending on the pacing site^17^; therefore, it is possible that passively-activated segments cannot be determined entirely by CS pacing alone even with HD mapping. Another reason could be the inability to create a complete transmural lesion at the directly-activated segments due to the limitations of the current ablation technology. However, in order to evaluate the formation of durable lesions, what can be done is to perform a second session to see whether there are gaps in the ablation lines. And the rate of recurrence might not necessarily be associated with the incompleteness of durable lesions. The last possible reason may be the various atrial fiber orientations at the PVs, which may mask or appear like passively-activated segments.

The limitations of our study included the following. First, our study was a single center, small prospective study, and based on our study results, a large, randomized study is warranted. Second, the activation pattern of the PV antrum was investigated only under CS pacing. Third, when the partial antral ablation was a failure, it is possible that the partial antral ablation could have been completed by creating a gap map and adding an additional RF application. Although gap mapping was not performed due to the procedural time limitation, additional useful information could have been provided by it. Fourth, we did not collect the detailed data about dormant conduction after the acute PVI. Finally, most of our study patients were paroxysmal AF patients, and whether the results of our study can be applied to persistent AF patients characterized by advanced atrial cardiomyopathy has not been investigated.

## Conclusion

HD mapping allows for evaluating the detailed activation patterns of the PVs, and it revealed the presence of passively-activated PV segments. A partial antral ablation of only directly-activated antral segments was feasible for achieving a PVI and reducing the chance of myocardial and possible collateral damage.

## Supplementary Information

Supplementary Information.

Supplementary Figure 1.

Supplementary Figure 2.

Supplementary Video 1.

Supplementary Video 2.

Supplementary Video 3.

Supplementary Video 4.
