# Supplementary material for: High density mapping guided partial antral ablation for a pulmonary vein isolation
Source: Sci Rep. 2021 Aug 16;11:16563. doi: 10.1038/s41598-021-96004-4 (PMC8367962; doi:10.1038/s41598-021-96004-4)
Supplement: Supplementary file 1 — Supplementary Information. [file 41598_2021_96004_MOESM1_ESM.docx]

**Supplementary contents legends**

1. Supplementary Figure 1. Example of LIPV activation mapping (Time difference between the earliest activated segment and the latest activated segment: 43ms)

2. Supplementary Video 1. Example of “direct activation pattern” of PV – focused on LSPV

3. Supplementary Video 2A. Example of presence of “passively activated segment (L3, L4 segment) of PV from adjacent PV segment” – focused on LIPV

4. Supplementary Video 2B. Example of presence of “passively activated segment (R1, R8 segment) of PV from adjacent PV segment” – focused on RSPV

5. Supplementary Video 2C. Example of presence of “passively activated segment (R3, R4 segment) of PV from adjacent PV segment” – focused on RIPV

6. Supplementary Figure 2. 1-year atrial tachyarrhythmia free survival graph of enrolled patients.
