# Supplementary figures and images for: High density mapping guided partial antral ablation for a pulmonary vein isolation
Source: Sci Rep. 2021 Aug 16;11:16563. doi: 10.1038/s41598-021-96004-4 (PMC8367962; doi:10.1038/s41598-021-96004-4)

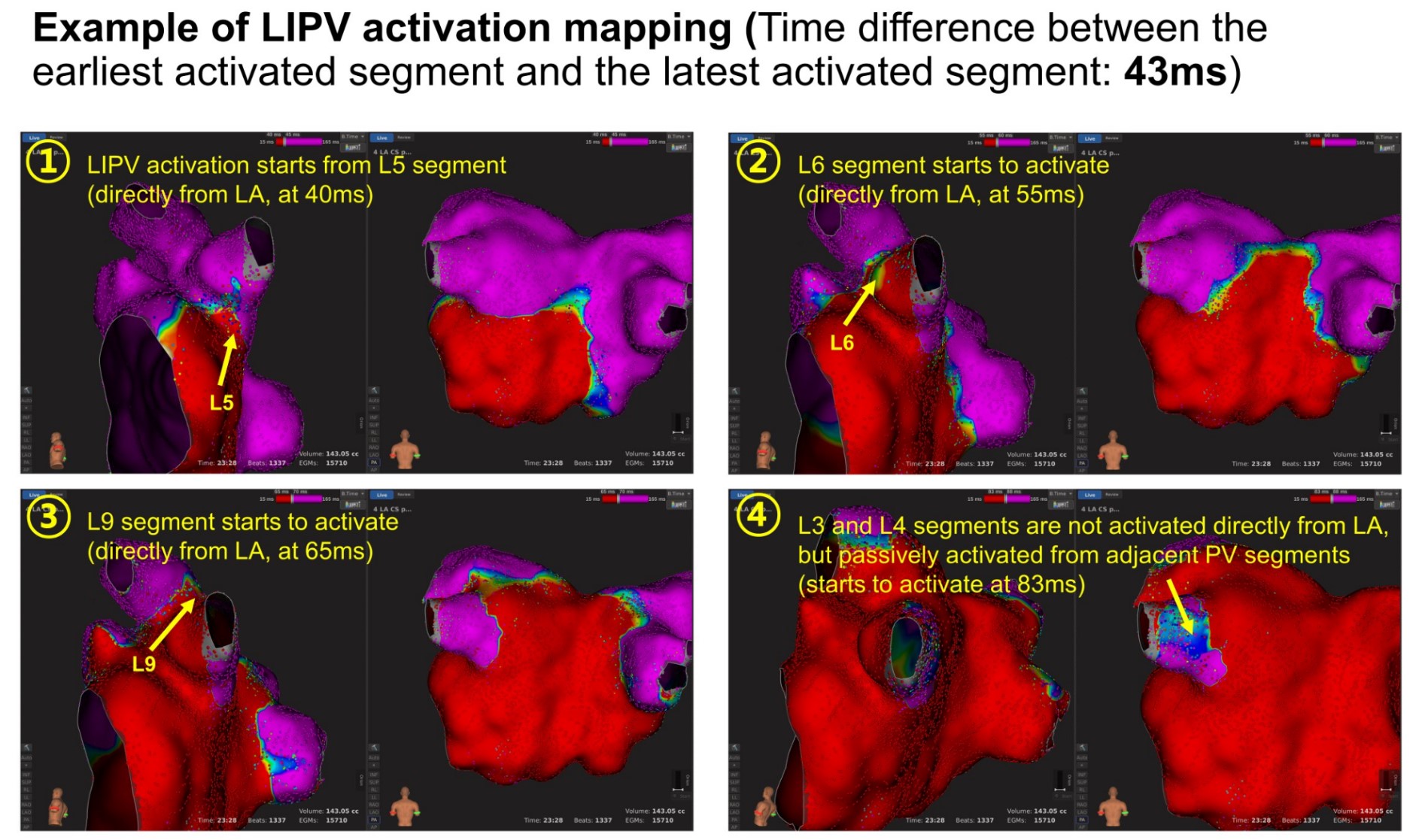

Supplement: Supplementary file 2 — Supplementary Figure 1. [file 41598_2021_96004_MOESM2_ESM.jpg]

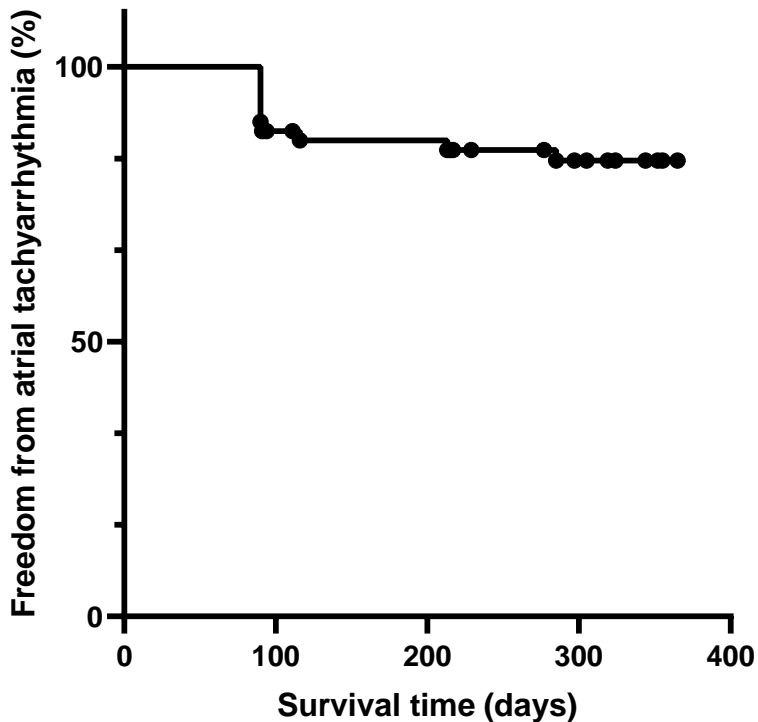

Supplement: Supplementary file 3 — Supplementary Figure 2. [file 41598_2021_96004_MOESM3_ESM.pdf]
